# The genome sequence of the Silver-spotted Skipper, *Hesperia comma *(Linnaeus, 1758)

**DOI:** 10.12688/wellcomeopenres.19762.1

**Published:** 2023-10-12

**Authors:** Konrad Lohse, Roger Vila, Alex Hayward, Dominik R. Laetsch, Eric Toro-Delgado, Wellcome Sanger Institute Tree of Life programme, Wellcome Sanger Institute Scientific Operations: DNA Pipelines collective, Tree of Life Core Informatics collective, Darwin Tree of Life Consortium

**Affiliations:** 1 Institute of Ecology and Evolution, The University of Edinburgh, Edinburgh, Scotland, UK; 2 Institut de Biologia Evolutiva, CSIC - Universitat Pompeu Fabra, Barcelona, Spain; 3 College of Life and Environmental Sciences, University of Exeter, Exeter, England, UK

**Keywords:** Hesperia comma, Silver-spotted Skipper, genome sequence, chromosomal, Lepidoptera

## Abstract

We present a genome assembly from an individual female *Hesperia comma* (the Silver-spotted Skipper; Arthropoda; Insecta; Lepidoptera; Hesperiidae). The genome sequence is 525.3 megabases in span. Most of the assembly is scaffolded into 29 chromosomal pseudomolecules, including the Z and W sex chromosomes. The mitochondrial genome has also been assembled and is 17.73 kilobases in length. Gene annotation of this assembly on Ensembl identified 18,725 protein coding genes.

## Species taxonomy

Eukaryota; Metazoa; Eumetazoa; Bilateria; Protostomia; Ecdysozoa; Panarthropoda; Arthropoda; Mandibulata; Pancrustacea; Hexapoda; Insecta; Dicondylia; Pterygota; Neoptera; Endopterygota; Amphiesmenoptera; Lepidoptera; Glossata; Neolepidoptera; Heteroneura; Ditrysia; Obtectomera; Hesperioidea; Hesperiidae; Hesperiinae; Hesperiini; *Hesperia*; *Hesperia comma* (Linnaeus, 1758) (NCBI:txid291688).

## Background

The Silver-spotted Skipper ( *Hesperia comma*) is a butterfly of the family Hesperiidae with a Holarctic distribution. It can be found throughout most of Europe, Asia and North America, as well as north Africa ( Tolman & Lewington, 1997). In the UK, this species is rare, and restricted to southern England.

*Hesperia comma* receives its common name by the appearance of the underside of its hindwings, which present two series of white lunules, one discal and another postdiscal, over a greenish background. The upper side of the wings is pale orange with a wide, dark brown margin with orange to pale yellow postdiscal spots. The species is sexually dimorphic, and males can be distinguished by the presence of a wide, black sex brand right below the discal cell of the forewing, which is absent in females.

This thermophilous species inhabits open areas, mainly chalk grasslands with sparse and short vegetation, where its main hostplants, *Festuca ovina* and *F. liviensis*, can be found. Females lay eggs single near the tip of leaf blades, with a preference for small tufts (usually no more than 5cm tall), adjacent to bare ground and not nibbled by grazing animals ( Davies *et al.*, 2005). *Hesperia comma* is univoltine, with a flight period from July to September, peaking in mid-August. However, in Alaska two seasons are needed to complete the development. It overwinters as egg or larva ( Tolman & Lewington, 1997).

Males are highly territorial, and the species has a relatively high dispersal capacity. Nevertheless, substantial genetic structure is documented at global scale ( Dapporto *et al.*, 2022; Forister *et al.*, 2004). While *H. comma* is listed as Least Concern in the European Red List of Butterflies ( Van Swaay *et al.*, 2010), it is classified as Vulnerable in the UK Red List ( Fox *et al.*, 2022). The species experienced a decline in the UK due to habitat loss and was reduced to 70 populations by 1982, but has subsequently recovered by an increase in suitable habitat due to grazing management, recovery of rabbit populations and climate change ( Davies *et al.*, 2005).

The species has been reported as having 28 chromosome pairs ( de Lesse, 1970; Larsen, 1975). Here we present a chromosomally complete genome sequence for *Hesperia comma*, based on one female specimen from Szöc, Hungary.

## Genome sequence report

**Figure 1.**
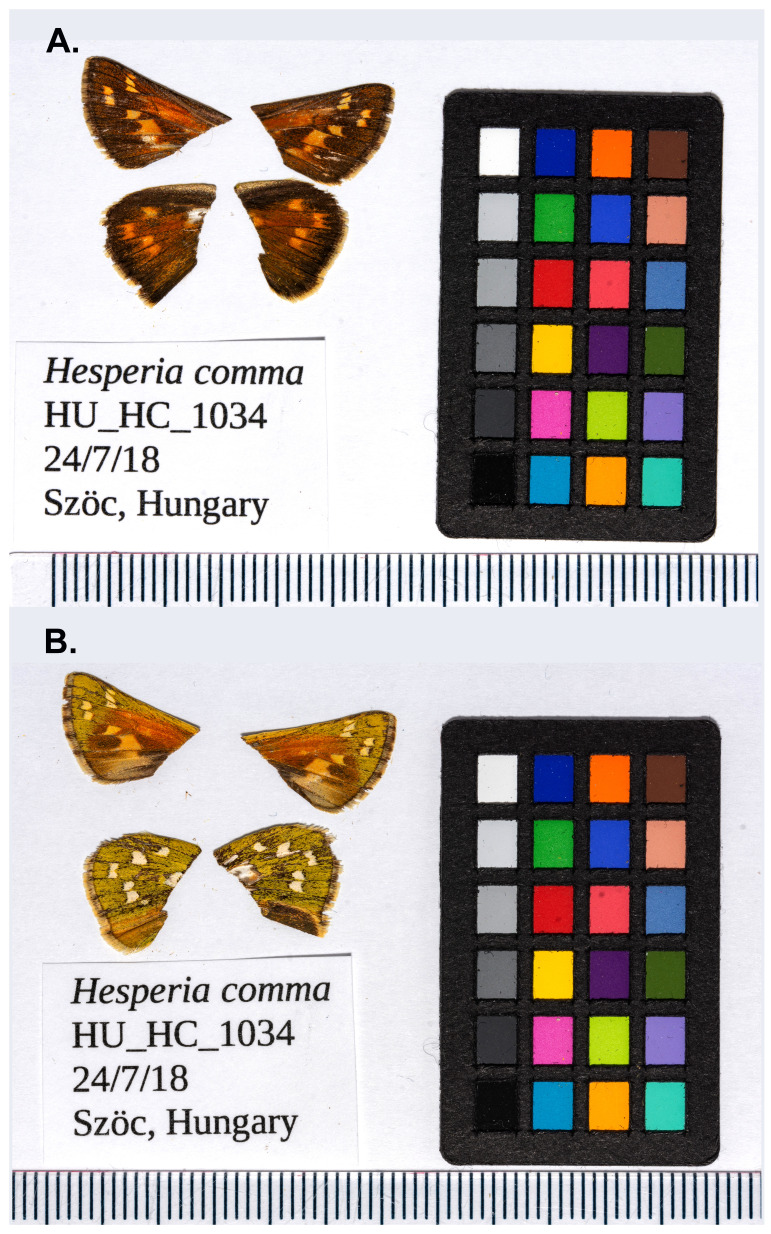
Forewings and hindwings of *Hesperia comma* (HU_HC_1034, ilHesComm1) specimen from Szöc, Hungary, from which the genome was sequenced. ( **A**) Dorsal surface view of wings ( **B**) Ventral surface view of wings.

The genome was sequenced from one female *Hesperia comma* ( Figure 1) collected from Szöc, Hungary (47.02, 17.51). A total of 28-fold coverage in Pacific Biosciences single-molecule HiFi long reads and 75-fold coverage in 10X Genomics read clouds was generated. Primary assembly contigs were scaffolded with chromosome conformation Hi-C data. Manual assembly curation corrected 105 missing joins or mis-joins and removed 30 haplotypic duplications, reducing the assembly length by 0.86% and the scaffold number by 51.85%, and increasing the scaffold N50 by 8.5%.

**Figure 2.**
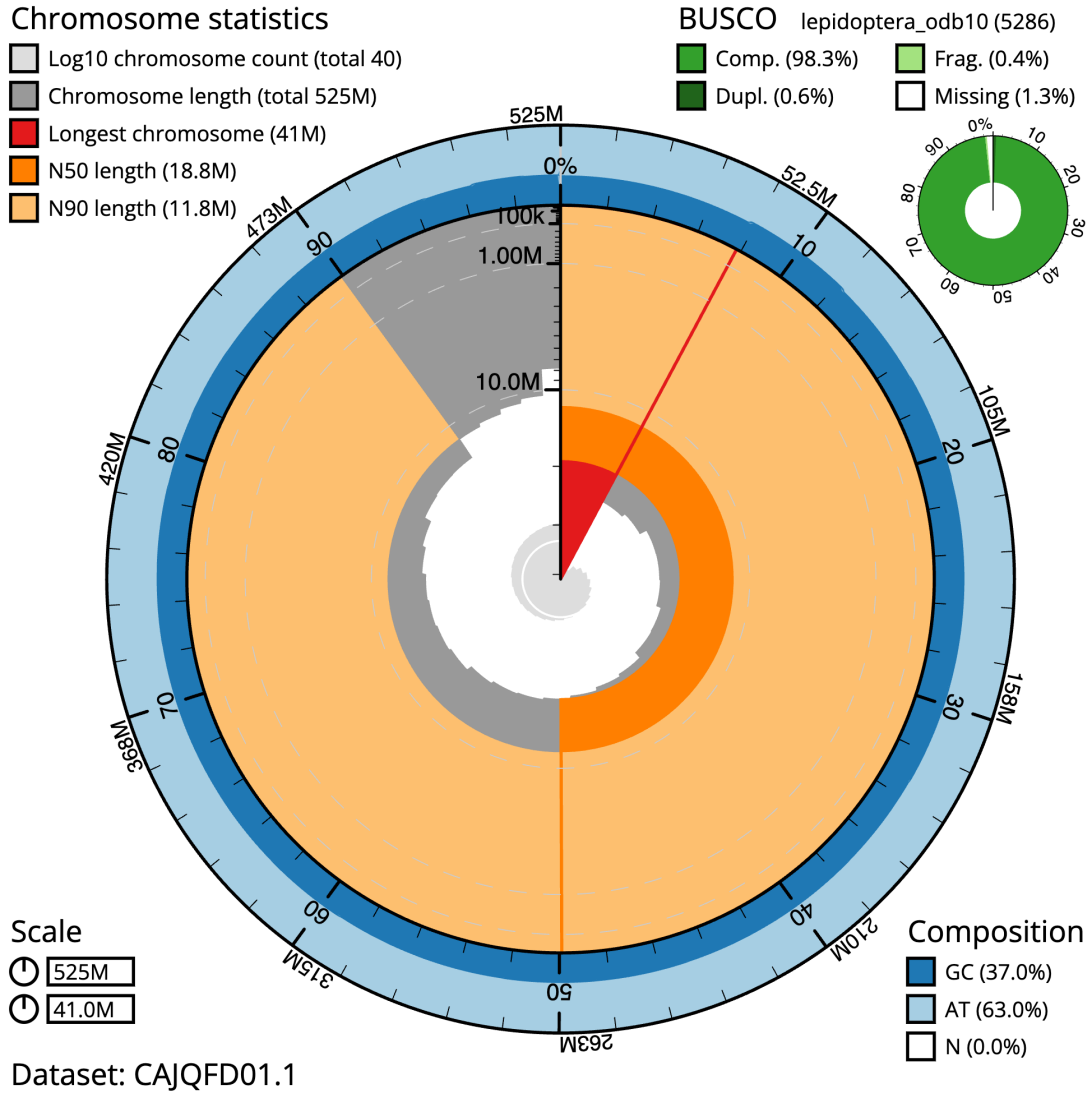
Genome assembly of *Hesperia comma*, ilHesComm1.1: metrics. The BlobToolKit Snailplot shows N50 metrics and BUSCO gene completeness. The main plot is divided into 1,000 size-ordered bins around the circumference with each bin representing 0.1% of the 525,353,014 bp assembly. The distribution of scaffold lengths is shown in dark grey with the plot radius scaled to the longest scaffold present in the assembly (41,004,304 bp, shown in red). Orange and pale-orange arcs show the N50 and N90 scaffold lengths (18,810,678 and 11,815,656 bp), respectively. The pale grey spiral shows the cumulative scaffold count on a log scale with white scale lines showing successive orders of magnitude. The blue and pale-blue area around the outside of the plot shows the distribution of GC, AT and N percentages in the same bins as the inner plot. A summary of complete, fragmented, duplicated and missing BUSCO genes in the lepidoptera_odb10 set is shown in the top right. An interactive version of this figure is available at https://blobtoolkit.genomehubs.org/view/ilHesComm1.1/dataset/CAJQFD01.1/snail.

The final assembly has a total length of 525.3 Mb in 39 sequence scaffolds with a scaffold N50 of 18.8 Mb ( Table 1). Most (99.96%) of the assembly sequence was assigned to 29 chromosomal-level scaffolds, representing 27 autosomes and the Z and W sex chromosomes. Chromosome-scale scaffolds confirmed by the Hi-C data are named in order of size ( Figure 2– Figure 5; Table 2). While not fully phased, the assembly deposited is of one haplotype. Contigs corresponding to the second haplotype have also been deposited. The mitochondrial genome was also assembled and can be found as a contig within the multifasta file of the genome submission.

**Table 1.** Genome data for *Hesperia comma*, ilHesComm1.1.

Project accession data		
Assembly identifier	ilHesComm1.1	
Species	*Hesperia comma*	
Specimen	ilHesComm1	
NCBI taxonomy ID	291688	
BioProject	PRJEB43800	
BioSample ID	SAMEA7523119	
Isolate information	ilHesComm1, female: whole organism (DNA sequencing and Hi-C scaffolding)	
Assembly metrics *		*Benchmark*
Consensus quality (QV)	56.9	*≥ 50*
*k*-mer completeness	99.99%	*≥ 95%*
BUSCO **	C:98.3%[S:97.7%,D:0.6%], F:0.4%,M:1.3%,n:5,286	*C ≥ 95%*
Percentage of assembly mapped to chromosomes	99.96%	*≥ 95%*
Sex chromosomes	W and Z chromosomes	*localised homologous pairs*
Organelles	Mitochondrial genome assembled	*complete single alleles*
Raw data accessions		
PacificBiosciences SEQUEL II	ERR6565940	
10X Genomics Illumina	ERR6054626–ERR6054629	
Hi-C Illumina	ERR6054630	
Genome assembly		
Assembly accession	GCA_905404135.1	
*Accession of alternate haplotype*	GCA_905404245.1	
Span (Mb)	525.3	
Number of contigs	125	
Contig N50 length (Mb)	10.3	
Number of scaffolds	39	
Scaffold N50 length (Mb)	18.8	
Longest scaffold (Mb)	41.0	
Genome annotation		
Number of protein-coding genes	18,725	
Number of gene transcripts	18,967	

* Assembly metric benchmarks are adapted from column VGP-2020 of “Table 1: Proposed standards and metrics for defining genome assembly quality” from ( Rhie *et al.*, 2021). ** BUSCO scores based on the lepidoptera_odb10 BUSCO set using v5.3.2. C = complete [S = single copy, D = duplicated], F = fragmented, M = missing, n = number of orthologues in comparison. A full set of BUSCO scores is available at https://blobtoolkit.genomehubs.org/view/ilHesComm1.1/dataset/CAJQFD01.1/busco.

**Figure 3.**
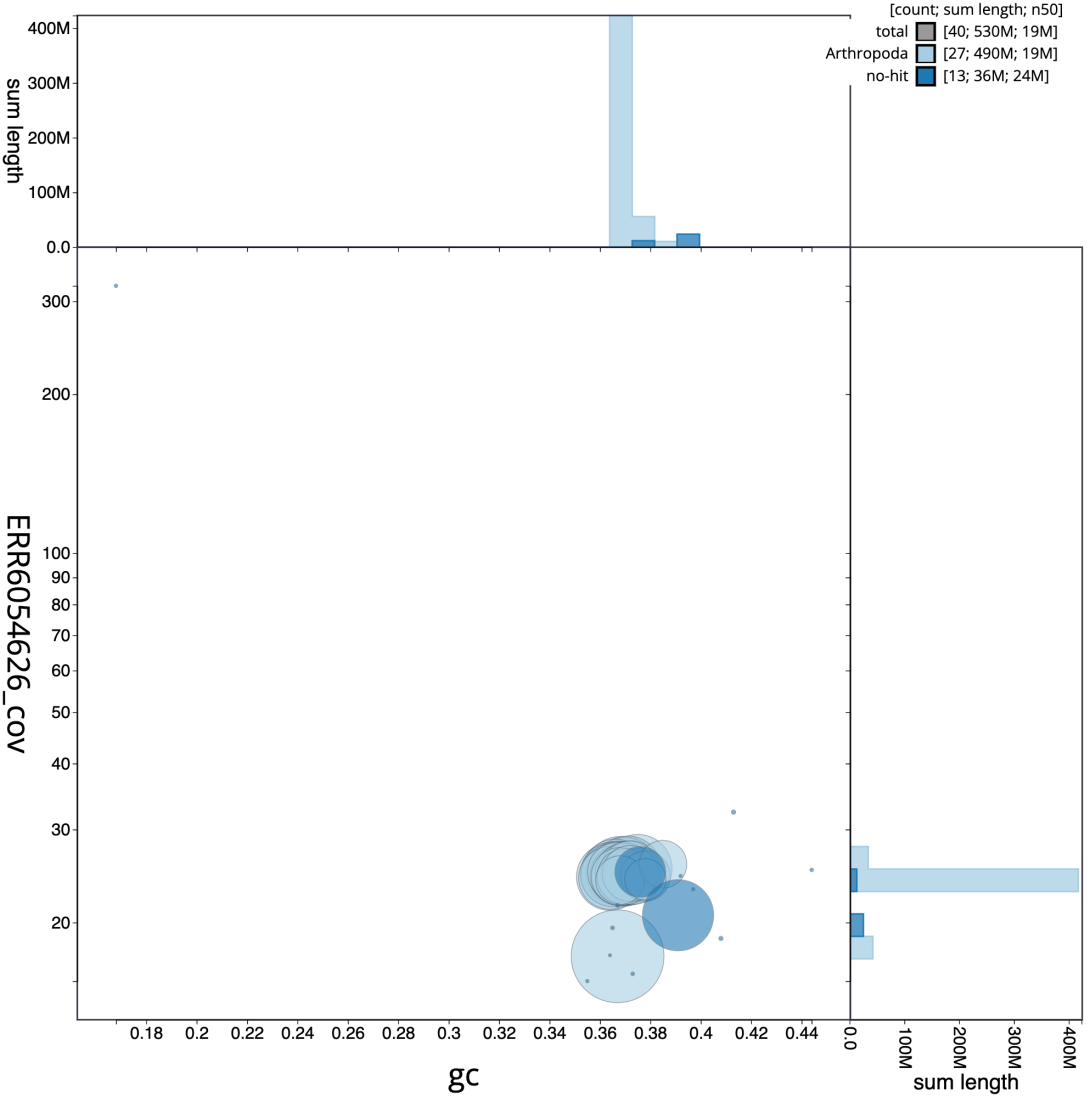
Genome assembly of *Hesperia comma*, ilHesComm1.1: BlobToolKit GC-coverage plot. Scaffolds are coloured by phylum. Circles are sized in proportion to scaffold length. Histograms show the distribution of scaffold length sum along each axis. An interactive version of this figure is available at https://blobtoolkit.genomehubs.org/view/ilHesComm1.1/dataset/CAJQFD01.1/blob.

**Figure 4.**
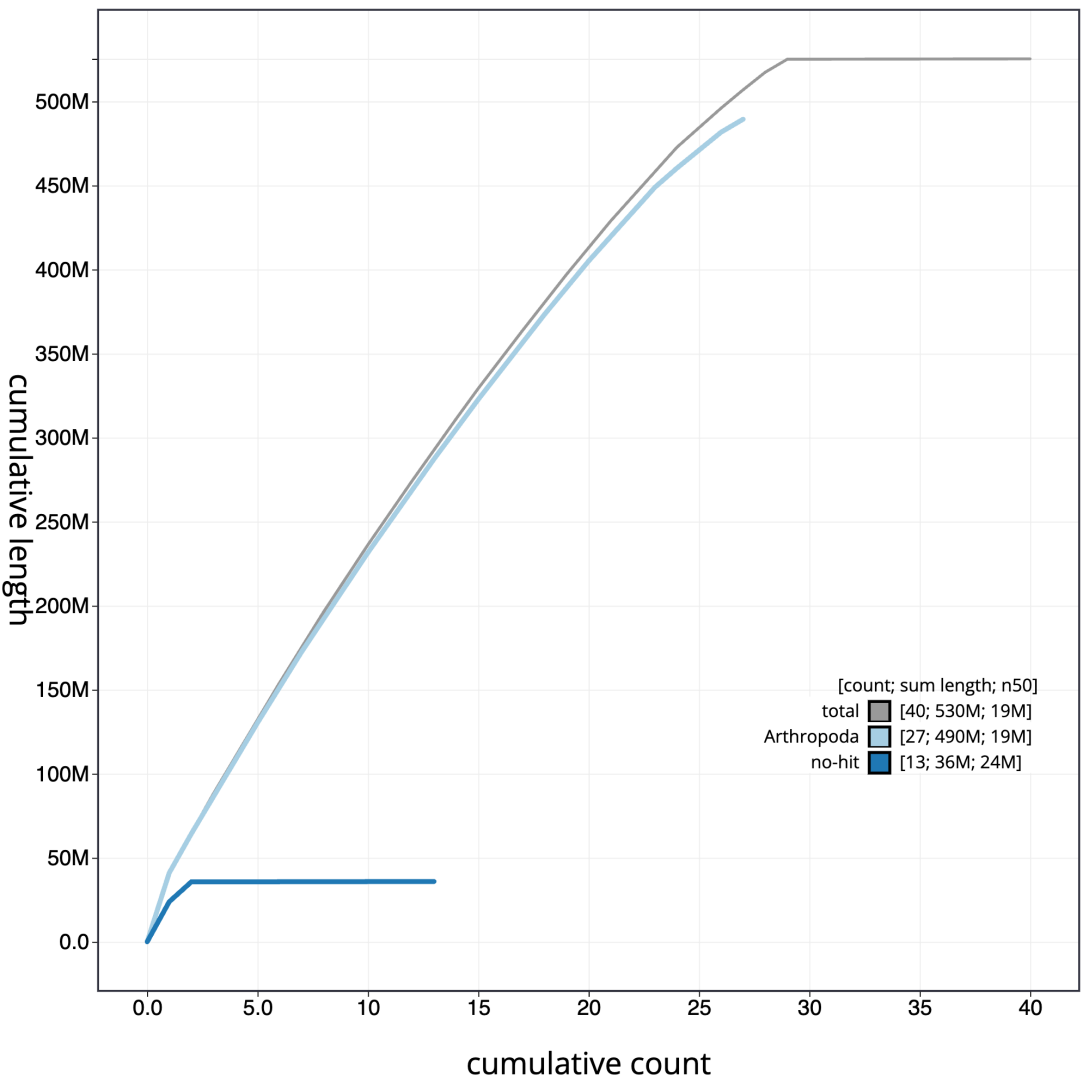
Genome assembly of *Hesperia comma*, ilHesComm1.1: BlobToolKit cumulative sequence plot. The grey line shows cumulative length for all scaffolds. Coloured lines show cumulative lengths of scaffolds assigned to each phylum using the buscogenes taxrule. An interactive version of this figure is available at https://blobtoolkit.genomehubs.org/view/ilHesComm1.1/dataset/CAJQFD01.1/cumulative.

**Figure 5.**
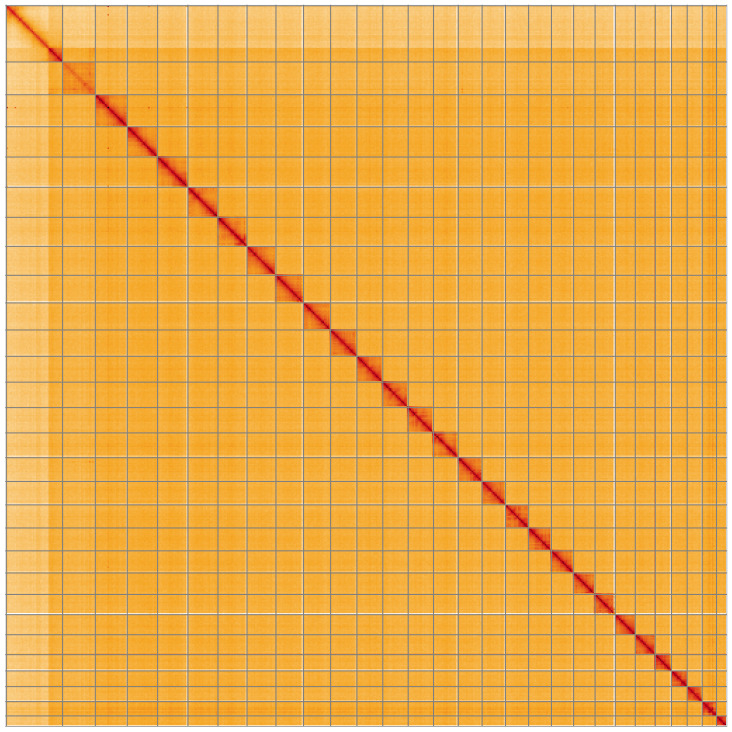
Genome assembly of *Hesperia comma*, ilHesComm1.1: Hi-C contact map of the ilHesComm1.1 assembly, visualised using HiGlass. Chromosomes are shown in order of size from left to right and top to bottom. An interactive version of this figure may be viewed at https://genome-note-higlass.tol.sanger.ac.uk/l/?d=BYtFurgBQWmecDExY_6qqw.

**Table 2.** Chromosomal pseudomolecules in the genome assembly of *Hesperia comma*, ilHesComm1.

INSDC accession	Chromosome	Length (Mb)	GC%
FR990014.1	1	23.28	37.0
FR990015.1	2	22.08	37.5
FR990016.1	3	22.04	37.0
FR990017.1	4	21.93	37.0
FR990018.1	5	21.18	37.0
FR990019.1	6	21.08	36.5
FR990020.1	7	20.06	36.5
FR990021.1	8	19.7	36.5
FR990022.1	9	19.21	36.5
FR990023.1	10	18.81	37.0
FR990024.1	11	18.55	36.5
FR990025.1	12	18.42	36.5
FR990026.1	13	18.06	37.0
FR990027.1	14	17.32	37.0
FR990028.1	15	17.04	37.0
FR990029.1	16	16.84	36.5
FR990030.1	17	16.65	37.0
FR990031.1	18	16.11	37.0
FR990032.1	19	15.67	37.0
FR990033.1	20	14.72	37.5
FR990034.1	21	14.58	37.0
FR990035.1	22	14.54	37.0
FR990036.1	23	11.82	37.5
FR990037.1	24	11.45	38.0
FR990038.1	25	10.92	37.0
FR990039.1	26	10.45	38.5
FR990040.1	27	7.73	38.0
FR990013.1	W	23.86	39.0
FR990012.1	Z	41.0	36.5
FR990041.1	MT	0.02	17.0

The estimated Quality Value (QV) of the final assembly is 56.9 with *k*-mer completeness of 99.99%, and the assembly has a BUSCO v5.3.2 completeness of 98.3% (single = 97.7%, duplicated = 0.6%), using the lepidoptera_odb10 reference set ( *n* = 5,286).

Metadata for specimens, spectral estimates, sequencing runs, contaminants and pre-curation assembly statistics can be found at https://links.tol.sanger.ac.uk/species/291688.

## Genome annotation report

The *Hesperia comma* genome assembly (GCA_905404135.1) was annotated using the Ensembl rapid annotation pipeline ( Table 1; https://rapid.ensembl.org/Hesperia_comma_GCA_905404135.1/Info/Index). The resulting annotation includes 18,967 transcribed mRNAs from 18,725 protein-coding genes.

## Methods

### Sample acquisition and nucleic acid extraction

A female *Hesperia comma* (ilHesComm1) was collected from Szöc, Hungary (latitude 47.02, longitude 17.51) on 2018-07-24 using a net. The specimen was collected by Konrad Lohse and Dominik Laetsch (University of Edinburgh) and Alex Hayward (University of Exeter), and identified by Konrad Lohse. The specimen was then frozen from live in a dry shipper.

DNA was extracted at the Tree of Life laboratory, Wellcome Sanger Institute (WSI). The ilHesComm1 sample was weighed and dissected on dry ice with tissue set aside for Hi-C sequencing. Tissue from the whole organism was disrupted using a Nippi Powermasher fitted with a BioMasher pestle. High molecular weight (HMW) DNA was extracted using the Qiagen MagAttract HMW DNA extraction kit. Low molecular weight DNA was removed from a 20 ng aliquot of extracted DNA using the 0.8X AMpure XP purification kit prior to 10X Chromium sequencing; a minimum of 50 ng DNA was submitted for 10X sequencing. HMW DNA was sheared into an average fragment size of 12–20 kb in a Megaruptor 3 system with speed setting 30. Sheared DNA was purified by solid-phase reversible immobilisation using AMPure PB beads with a 1.8X ratio of beads to sample to remove the shorter fragments and concentrate the DNA sample. The concentration of the sheared and purified DNA was assessed using a Nanodrop spectrophotometer and Qubit Fluorometer and Qubit dsDNA High Sensitivity Assay kit. Fragment size distribution was evaluated by running the sample on the FemtoPulse system.

### Sequencing

Pacific Biosciences HiFi circular consensus and 10X Genomics read cloud DNA sequencing libraries were constructed according to the manufacturers’ instructions. DNA sequencing was performed by the Scientific Operations core at the WSI on Pacific Biosciences SEQUEL II (HiFi) and HiSeq X Ten (10X) instruments. Hi-C data were also generated from tissue of ilHesComm1 using the Arima2 kit and sequenced on the Illumina NovaSeq 6000 instrument.

### Genome assembly, curation and evaluation

Assembly was carried out with Hifiasm ( Cheng *et al.*, 2021) and haplotypic duplication was identified and removed with purge_dups ( Guan *et al.*, 2020). One round of polishing was performed by aligning 10X Genomics read data to the assembly with Long Ranger ALIGN, calling variants with FreeBayes ( Garrison & Marth, 2012). The assembly was then scaffolded with Hi-C data ( Rao *et al.*, 2014) using SALSA2 ( Ghurye *et al.*, 2019). The assembly was checked for contamination and corrected using the gEVAL system ( Chow *et al.*, 2016) as described previously ( Howe *et al.*, 2021). Manual curation was performed using gEVAL, HiGlass ( Kerpedjiev *et al.*, 2018) and Pretext ( Harry, 2022). The mitochondrial genome was assembled using MitoHiFi ( Uliano-Silva *et al.*, 2022), which runs MitoFinder ( Allio *et al.*, 2020) or MITOS ( Bernt *et al.*, 2013) and uses these annotations to select the final mitochondrial contig and to ensure the general quality of the sequence.

A Hi-C map for the final assembly was produced using bwa-mem2 ( Vasimuddin *et al.*, 2019) in the Cooler file format ( Abdennur & Mirny, 2020). To assess the assembly metrics, the *k*-mer completeness and QV consensus quality values were calculated in Merqury ( Rhie *et al.*, 2020). This work was done using Nextflow ( Di Tommaso *et al.*, 2017) DSL2 pipelines “sanger-tol/readmapping” ( Surana *et al.*, 2023a) and “sanger-tol/genomenote” ( Surana *et al.*, 2023b). The genome was analysed within the BlobToolKit environment ( Challis *et al.*, 2020) and BUSCO scores ( Manni *et al.*, 2021; Simão *et al.*, 2015) were calculated.

Table 3 contains a list of relevant software tool versions and sources.

**Table 3.** Software tools: versions and sources.

Software tool	Version	Source
BlobToolKit	4.1.5	https://github.com/blobtoolkit/blobtoolkit
BUSCO	5.3.2	https://gitlab.com/ezlab/busco
FreeBayes	1.3.1-17-gaa2ace8	https://github.com/freebayes/freebayes
gEVAL	N/A	https://geval.org.uk/
Hifiasm	0.12	https://github.com/chhylp123/hifiasm
HiGlass	1.11.6	https://github.com/higlass/higlass
Long Ranger ALIGN	2.2.2	https://support.10xgenomics.com/genome-exome/ software/pipelines/latest/advanced/other-pipelines
Merqury	MerquryFK	https://github.com/thegenemyers/MERQURY.FK
MitoHiFi	2	https://github.com/marcelauliano/MitoHiFi
PretextView	0.2	https://github.com/wtsi-hpag/PretextView
purge_dups	1.2.3	https://github.com/dfguan/purge_dups
SALSA	2.2	https://github.com/salsa-rs/salsa
sanger-tol/genomenote	v1.0	https://github.com/sanger-tol/genomenote
sanger-tol/readmapping	1.1.0	https://github.com/sanger-tol/readmapping/tree/1.1.0

### Genome annotation

The BRAKER2 pipeline ( Brůna *et al.*, 2021) was used in the default protein mode to generate annotation for the *Hesperia comma* assembly (GCA_905404135.1) in Ensembl Rapid Release.

### Wellcome Sanger Institute – Legal and Governance

The materials that have contributed to this genome note have been supplied by a Tree of Life collaborator. The Wellcome Sanger Institute employs a process whereby due diligence is carried out proportionate to the nature of the materials themselves, and the circumstances under which they have been/are to be collected and provided for use. The purpose of this is to address and mitigate any potential legal and/or ethical implications of receipt and use of the materials as part of the research project, and to ensure that in doing so we align with best practice wherever possible. The overarching areas of consideration are:

•   Ethical review of provenance and sourcing of the material

•   Legality of collection, transfer and use (national and international)

Each transfer of samples is undertaken according to a Research Collaboration Agreement or Material Transfer Agreement entered into by the Tree of Life collaborator, Genome Research Limited (operating as the Wellcome Sanger Institute) and in some circumstances other Tree of Life collaborators.

## Data Availability

European Nucleotide Archive: *Hesperia comma* (common branded skipper). Accession number PRJEB43800; https://identifiers.org/ena.embl/PRJEB43800. ( Wellcome Sanger Institute, 2021) The genome sequence is released openly for reuse. The *Hesperia comma* genome sequencing initiative is part of the Darwin Tree of Life (DToL) project. All raw sequence data and the assembly have been deposited in INSDC databases. Raw data and assembly accession identifiers are reported in Table 1.

## Author information

Members of the Wellcome Sanger Institute Tree of Life programme are listed here: https://doi.org/10.5281/zenodo.4783585.

Members of Wellcome Sanger Institute Scientific Operations: DNA Pipelines collective are listed here: https://doi.org/10.5281/zenodo.4790455.

Members of the Tree of Life Core Informatics collective are listed here: https://doi.org/10.5281/zenodo.5013541.

Members of the Darwin Tree of Life Consortium are listed here: https://doi.org/10.5281/zenodo.4783558.
