# Supplementary material for: The genome sequence of the Silver-spotted Skipper, Hesperia comma (Linnaeus, 1758)
Source: Wellcome Open Res. 2023 Oct 12;8:457. [Version 1] doi: 10.12688/wellcomeopenres.19762.1 (PMC12859415; doi:10.12688/wellcomeopenres.19762.1)
Supplement: Peer review history

# Reviewer report: Reviewer response for version 1

**DOI:** 10.21956/wellcomeopenres.21888.r73259

**Published:** 2026-01-30

**Authors:** Alexander Kneubehl

**Affiliations:** 1 Department of Pediatrics, Baylor College of Medicine, Houston, TX, USA

Lohse et al. present here a genome sequence of the Silver-spotted Skipper. The genome was assembled into chromosomal pseudomolecules using PacBio HiFi, 10x Genomics, and Illumina HiC data. These data yielded 27 autosomes as well as the Z and W sex chromosomes. The authors present their data well and do a good job of describing their methods. The only deficiency is that they discuss this organism as being "Vulnerable" in the UK but then go on to sequence a specimen from Hungary where this species is listed as "Least Concern". They indicate that there is a substantial genetic structure on a global scale for this organism so it would have been interesting to sequence a UK specimen to preserve that genetic information. A bit more rationale on why a Hungarian specimen was selected would be useful here.

Are sufficient details of methods and materials provided to allow replication by others?

Yes

Is the rationale for creating the dataset(s) clearly described?

Partly

Are the datasets clearly presented in a useable and accessible format?

Yes

Are the protocols appropriate and is the work technically sound?

Yes

Reviewer Expertise:

Microbial genomics, arthropod genomics, comparative genomics, infectious diseases, vectorborne diseases

I confirm that I have read this submission and believe that I have an appropriate level of expertise to confirm that it is of an acceptable scientific standard.

# Reviewer report: Reviewer response for version 1

**DOI:** 10.21956/wellcomeopenres.21888.r73257

**Published:** 2024-02-20

**Authors:** José M Martín-Durán

**Affiliations:** 1 School of Biological and Behavioural Sciences, Queen Mary University of London, London, England, UK

Hesperia comma is a cosmopolitan butterfly that can be found in the southern part of England. This manuscript reports the reference genome of this species. As with other assemblies of the DToL project, the outcome of the sequencing efforts follows gold-standard principles and will be a valuable resource for the community working on diverse research fields, from evolutionary biology to ecology.

Are sufficient details of methods and materials provided to allow replication by others?

Yes

Is the rationale for creating the dataset(s) clearly described?

Yes

Are the datasets clearly presented in a useable and accessible format?

Yes

Are the protocols appropriate and is the work technically sound?

Yes

Reviewer Expertise:

Evolutionary biology, comparative genomics, developmental biology

I confirm that I have read this submission and believe that I have an appropriate level of expertise to confirm that it is of an acceptable scientific standard.
